# Overexpression of VEGF-C and MMP-9 predicts poor prognosis in Kazakh patients with esophageal squamous cell carcinoma

**DOI:** 10.7717/peerj.8182

**Published:** 2019-12-03

**Authors:** Jiangfen Li, Yufang Xie, Xueli Wang, Chenhao Jiang, Xin Yuan, Anzhi Zhang, Chunxia Liu, Lijuan Pang, Feng Li, Jianming Hu

**Affiliations:** 1 Department of Pathology and Key Laboratory for Xinjiang Endemic and Ethnic Diseases (Ministry of Education)/Department of Pathology, the First Affiliated Hospital, Shihezi University School of Medicine, Xinjiang, China; 2 Capital Medical University, Department of Pathology, Beijing Chaoyang Hospital, Beijing, China

**Keywords:** Kazakh, Esophageal cancer, Esophageal squamous cell carcinoma, MMP-9, VEGF-C

## Abstract

Vascular endothelial growth factor (VEGF*)* and Matrix metalloproteinases (MMPs) are believed to participate in infiltration of tumors. High mortality of esophageal squamous cell carcinoma (ESCC) related to its primary infiltration; however, it is not clear whether the expression of VEGF and MMPs is involved in this process. Screening of The Cancer Genome Atlas (TCGA) database showed that among the *VEGF* family and *MMP9*, *VEGF-A*, *VEGF-C,* and *MMP-9* mRNA were overexpression in ESCC. This result was verified using the Oncomine database and in Kazakh patients with ESCC. Overexpression of *VEGF-C* and *MMP-9* and positive association with advanced esophageal cancer and invading ESCC cells (Gene Expression Omnibus (GEO): GSE21293). Immunohistochemical staining revealed that VEGF-C and MMP-9 were overexpressed in Kazakh ESCCs. VEGF-C expression was related to invasive depth, tumor-node-metastasis (TNM) staging, lymphatic, and lymph node metastasis of ESCC. The linear association between them was further confirmed in TCGA database and the specimens from Kazakh patients with ESCC. Patients with both proteins expression had tumors with greater aggressiveness, suffered from poor prognosis compared with patients who did not express either protein or expressed protein alone. Both proteins expression predicted high invasiveness of ESCC, which is related to worse prognosis of Kazakh ESCCs.

## Introduction

Esophageal cancer (EC) is an aggressive malignancy originating in the gastrointestinal tract. EC has the eighth highest tumor incidence and is the sixth frequent lead to tumor death on a global scale (Allemani et al., 2015). For China, esophageal squamous cell carcinoma (ESCC) is the main pathological subtypes of EC. Morbidity from ESCC in the Kazakh population from Xinjiang (China) is far more than other populations (Zheng et al., 2010). The symptoms of EC can remain hidden and the disease progresses rapidly, therefore, the clinical efficacy of treatments and patient prognosis are poor, resulting in a five-year survival rate of only 10% (Ekman et al., 2008). High mortality of ESCC closely related to its primary infiltration (Cools-Lartigue, Spicer & Ferri, 2015). Therefore, the determination of the metastatic mechanism is necessary and will provide the basis for the development of novel therapies to treat ESCC.

Asahara et al. (1999) reported that Vascular endothelial growth factor (VEGF) could regulate endothelial progenitor cells (EPC), induce the differentiation of EPCs, and promote angiogenesis. The VEGF family have a range of members, which play different roles in promoting tumor angiogenesis (Ferrara, Gerber & LeCouter, 2003). VEGF-C is considered a special lymphangiogenesis factor because it could promote lymphatic endothelial cells’ formation and differentiation (Karkkainen et al., 2004). The expression level of VEGF-C is related to lymph vessel number, and it thought to be an independent prognostic element in multiple malignant tumors (Kitadai et al., 2001; Skobe et al., 2001).

Matrix metalloproteinases (MMPs) are composed of more than 20 different members (Klein & Bischoff, 2011). They promote vascularization, ruin tissue structure, and basal membranes, thus allowing tumor infiltration (Kerkelä & Saarialho-Kere, 2010). Forsyth investigated MMP-2, MMP-9, and MT1-MMPs’ function in the progression of glioma, in which MMP-9 was mainly participated in vascularization and remodeling (Forsyth et al., 1999). MMP-9 can degrade collagen IV in tumor tissue, promoting the vascularization and infiltration of EC (Magdalena et al., 2012). In our research at a previous time, MMP-9 was overexpression in Kazakh ESCCs. It was related to a lot of invasive features (Hu et al., 2017). Notably, MMP-9 could promote the release of VEGF to promote tumor vascular formation (Bergers et al., 2000). Thus, interaction between them might be associated with ESCC in the Kazakh population.

MMP-9 and VEGF have been investigated as important factors related to invasion and metastasis in tumors (Zhao et al., 2008). However, which subtype of the VEGF family members is most closely related to Kazakh ESCC remains unclear, and no study have evaluated both proteins simultaneously. Accordingly, we detected the mRNA level of *VEGF/MMP-9* in EC utilizing TCGA and Oncomine databases, and Kazakh ESCCs, to identify the *VEGF* family member that has the most abnormal expression. Next, we explored the roles of *VEGF* family members and *MMP-9* in ESCC invasion. We subsequently studied the correlations between *VEGF* family members and *MMP-9* based on TCGA EC samples. Then, we detected VEGF-C/MMP-9 protein in Kazakh patients with ESCC, combined with the clinicopathological parameters of the patients. Finally, we investigated co-overexpression of their effect in Kazakh patients with ESCC.

## Materials & Methods

### Patients and specimens

All the patients were from the Kazakh national minority ethnic population and had been living in the Yili region of Xinjiang, China, where they experienced the same environmental exposure as the Chinese population. None of them had received radiotherapy or chemotherapy before surgery. Please refer to Table S1 for the characteristic of the patients.

Two senior pathologists did not know the clinicopathological information about the samples at all when they assessed, and they also judged the results entirely independently. If there were differences in opinion in the judgment results, a third pathologist would judge the samples again, and the opinions of the three pathologists would provide the final result. Data were collected and quantified as bewrited previously (Hu et al., 2017).

### Immunohistochemistry

To detect the expression of VEGF-C/MMP-9 proteins in Kazakh ESCCs, immunohistochemistry (IHC) was applied, then detected and quantified according to the methods described previously (Hu et al., 2017). The anti-VEGF-C monoclonal antibodies (mAbs) and anti-MMP9 mAbs (Santa Cruz, USA) were applied in this possess.

Two senior pathologists assessed the result. Positive IHC staining was assessed following Santa Cruz Biotechnology s’ instructions. The interpretation of the results is as bewrited previously (Hu et al., 2017).

### Bioinformatic analysis

To analyze the mRNA level of *VEGF*/*MMP*-*9* in EC, TCGA data were analyzed. Data were downloaded and analyzed from the UALCAN website (http://ualcan.path.uab.edu/analysis.html). It has 185 cases of EC and 11 cases of normal esophagus tissues (Chandrashekar et al., 2017). Microarray gene expression data from two different subtypes of ESCC and normal tissue were included in this study. Oncomine website (https://www.oncomine.org) data was also used in this process, from which we included two datasets: The Su Esophagus 2 dataset, which includes 53 ESCC samples and 53 normal samples; and The Kimchi Esophagus dataset, which includes eight esophageal adenocarcinoma (EAC) samples and eight normal samples. According to the online analysis function of the two databases, mRNA expression in two subtypes of EC was analyzed, when the *P*-value < 0.05, it is deemed significant.

To further explore the mRNA level of the *VEGF* family and *MMP*-*9* in different stages of EC, we used the GEPIA website (http://gePia.cancer-Pku.cn/). This database can analyze the expression of mRNA in different tumor stages based on TCGA microarray data (Tang et al., 2017). There were data for 182 cases of EC. The correlation between *VEGF* family member expression and *MMP*-*9* expression were also analyzed at GEPIA, *F* test ≥ 2.75, *P*-Value < 0.05 was deemed as obvious divergence.

GSE21293 from GEO (http://www.ncbi.nlm.nih.gov/geo), was brought into our research. The GSE21293 profile contained gene expression data of invading and non-invading ESCC cells. The differentially expressed mRNA were evaluated using the GEO2R (https://www.ncbi.nlm.nih.gov/geo/geo2r/), and the parameters were “adjusted *P*-value < 0.05, |log*FC*| ≥ 1.0”. The raw data were downloaded, and standardized data after log2 transformation of FPKM values were used. Finally, the standardized data were applied to calculate the mRNA level of the *VEGF* family and *MMP*-*9* in invading and non-invading ESCC cells; a *P*-Value < 0.05 was deemed to divergence obvious.

### RNA extraction and qRT-PCR

FFPE specimens were cut in 10-µm sections and 10 pieces of them were transferred into no enzyme Eppendorf tubes. First, we used xylene-ethanol to remove the paraffin from the FFPE tissue sections (Coura et al., 2005). Then, RNA was isolated from FFPE using the TRIzol reagent (Thermofisher, USA) in the light of the instructions. Finally, the RNA yield and quality was detected applying NanoDrop spectrophotometer (Thermofisher).

Next, cDNA was synthesized from the RNA samples applying SuperQuick RT MasterMix (CWBIO, Beijing, China). QRT-PCR was carried out with UltraSYBR Mixture (CWBIO), based on the manufacturer’s protocol. All gene expression values were calculated in relation to the expression of *ACTB* (encoding ß-actin) and the changes in mRNA expression were calculated by the 2^−ΔΔ*Ct*^ means (Livak & Schmittgen, 2001). The primer sequences are shown in Table S2.

### Statistical analysis

SPSS v.20.0 (IBM, USA) was used for statistical analysis of all data in Kazakh ESCCs. Student’s *t*-test and Nonparametric Test were applied to find the difference in mRNA and proteins expression between the ESCC and CAN sets. The Spearman method was applied to assess the relationship between each molecule. The relationship between proteins expression and clinical parameters were analyzed by ANOVA. Kaplan–Meier method and Cox proportional risk regression model was used to assessing patient outcomes, a *P*-Value < 0.05 was supposed to divergence significant.

## Results

### *VEGF* family members and *MMP-9* mRNA expression in EC and their correlations with progression of EC

**Figure 1:**
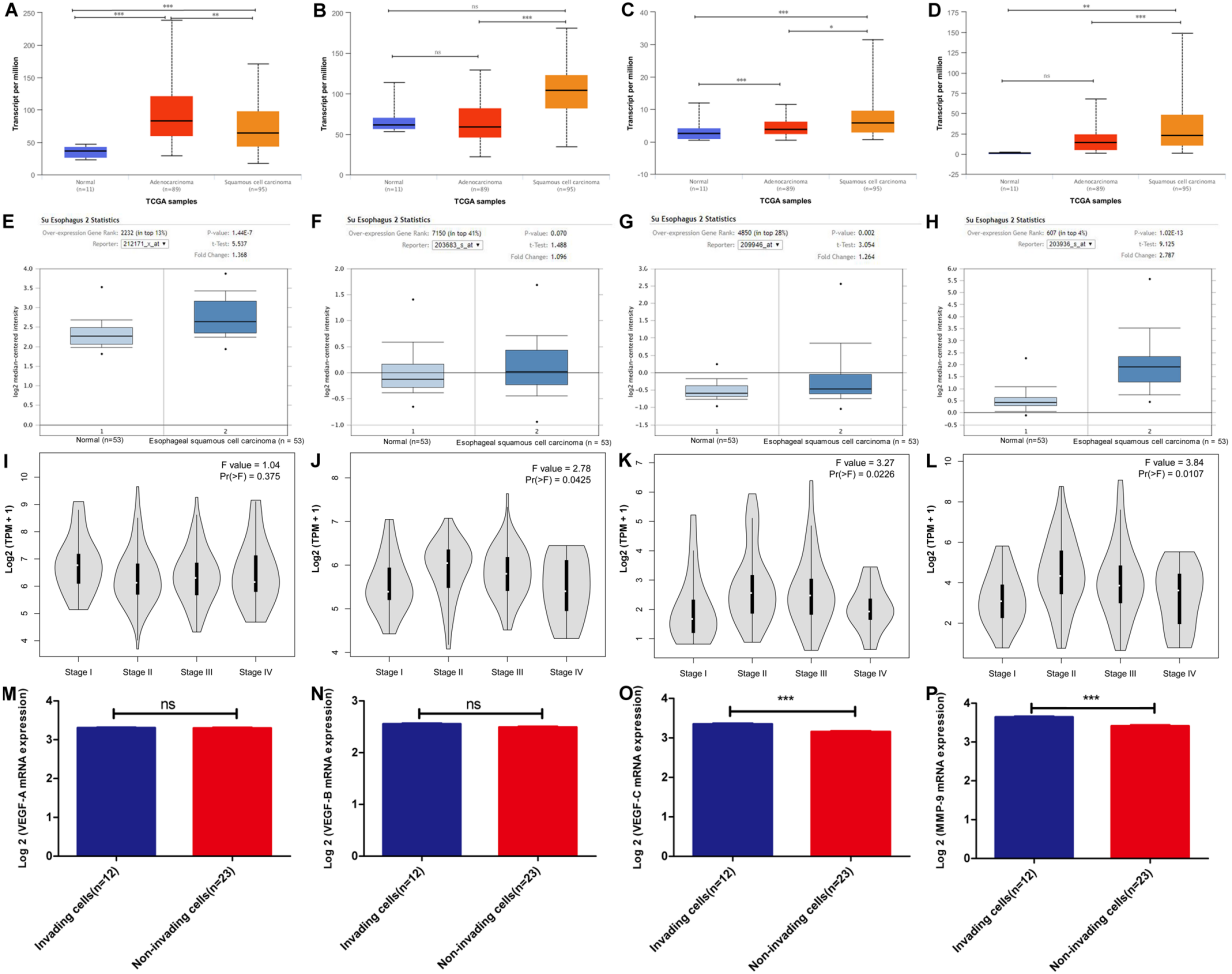
*VEGF* family members and *MMP-9* mRNA expression in esophageal cancer (EC) according to bioinformatics analysis. (A–D) The Ualcan database (including TCGA esophageal samples *n* = 195) was applied to analyze (A) *VEGF-A*, (B) *VEGF-B*, (C) *VEGF-C*, and (D) *MMP-9* mRNA expression in ESCC, EAC, and normal tissues. Rank sum test, ns *P* > 0.05, *P < 0.05, **P < 0.01, ***P < 0.001. (E–H) *VEGF* and *MMP-9* mRNA expression in ESCC tissues versus normal samples from Oncomine database. (E) *VEGF-A*, (F) *VEGF-B*, (G) *VEGF-C*, and (H) *MMP-9*. *n* = 106, Student’s *t*-test. (I–L) The mRNA expression of (I) *VEGF-A*, (J) *VEGF-B*, (K) *VEGF-C*, and (L) *MMP-9* in different pathological stages of EC based on the GEPIA database (including TCGA EC samples *n* = 182). Variance homogeneity test. (M–P) The microarray profiling (GEO, GSE37475) from GEO database were validated for the mRNA expression of (M) *VEGF-A*, (N) *VEGF-B*, (O) *VEGF-C*, and (P) *MMP-9* in the invading cells group (*n* = 12) relative to the non-invading cells group (*n* = 23). Differentially expressed genes enriched in invading cells were identified when the average gene expression ratio of invading cells/non-invading cells was ≥1.0-fold. Standardized data after log2 transformation of FPKM values of all genes was used. Student’s *t*-test, ns *P* > 0.05, *P < 0.05, **P < 0.01, ***P < 0.001.

Based on the TCGA esophagus samples, we analyzed the *VEGF* family members and *MMP-9* mRNA. As shown in Figs. 1A–1D, *VEGF-A* was predominantly and highly expressed in EAC tissues (Fig. 1A), whereas *VEGF-B* showed no abnormal expression in either EAC or ESCC tissues (Fig. 1B). Notably, compared with *VEGF-C* expression in EAC and normal tissues, it was highly expressed in ESCC (Fig. 1C). *MMP-9* was overexpressed in ESCC, but not in EAC, compared with that in normal tissues (Fig. 1D). To confirm the above results, Oncomine database analysis was carried out. Basically consistent with TCGA results (Figs. 1E–1H, Figs. S1A–S1D). *VEGF-C* mRNA was primarily overexpressed in ESCC, *VEGF-A* mRNA was primarily overexpressed in EAC, and *MMP-9* mRNA was highly expressed in both EC subtypes.

Pathological staging is an important index in tumor diagnosis for prognosis assessment and treatment planning (Gleason & Mellinger, 2017). Thus, we texted *VEGF* and *MMP-9* mRNA expression in different pathological stages of EC based on the TCGA samples, and a variance homogeneity test was carried out. The results showed that the correlation between *VEGF-A* (Fig. 1I) mRNA expression and EC pathological stage was not obvious, while the expression levels of *VEGF-B* (Fig. 1G), *VEGF-C* (Fig. 1K), and *MMP-9* mRNA (Fig. 1L) were associated with the EC pathological stage.

To determine the role of *VEGF* family members and *MMP-9* in ESCC invasion, we used a GEO microarray dataset (GSE21293), found that *MMP-9* (log FC = 1.82, *P* < 0.05) and *VEGF-C* mRNA (log FC = 1.27, *P* < 0.05) expression were significantly up-regulated in invading ESCC cells relative to the matched non-invading ESCC cells, while there was no significant up-regulation of *VEGF-A* mRNA and *VEGF-B* mRNA in invading ESCC cells (Figs. 1M–1P). Taken together, these data demonstrated *VEGF-C*/*MMP-9* are primarily overexpressed in ESCC, may mediate ESCC infiltration.

### *VEGF* family members and *MMP-9* mRNA expression in Kazakh patients with ESCC

**Figure 2:**
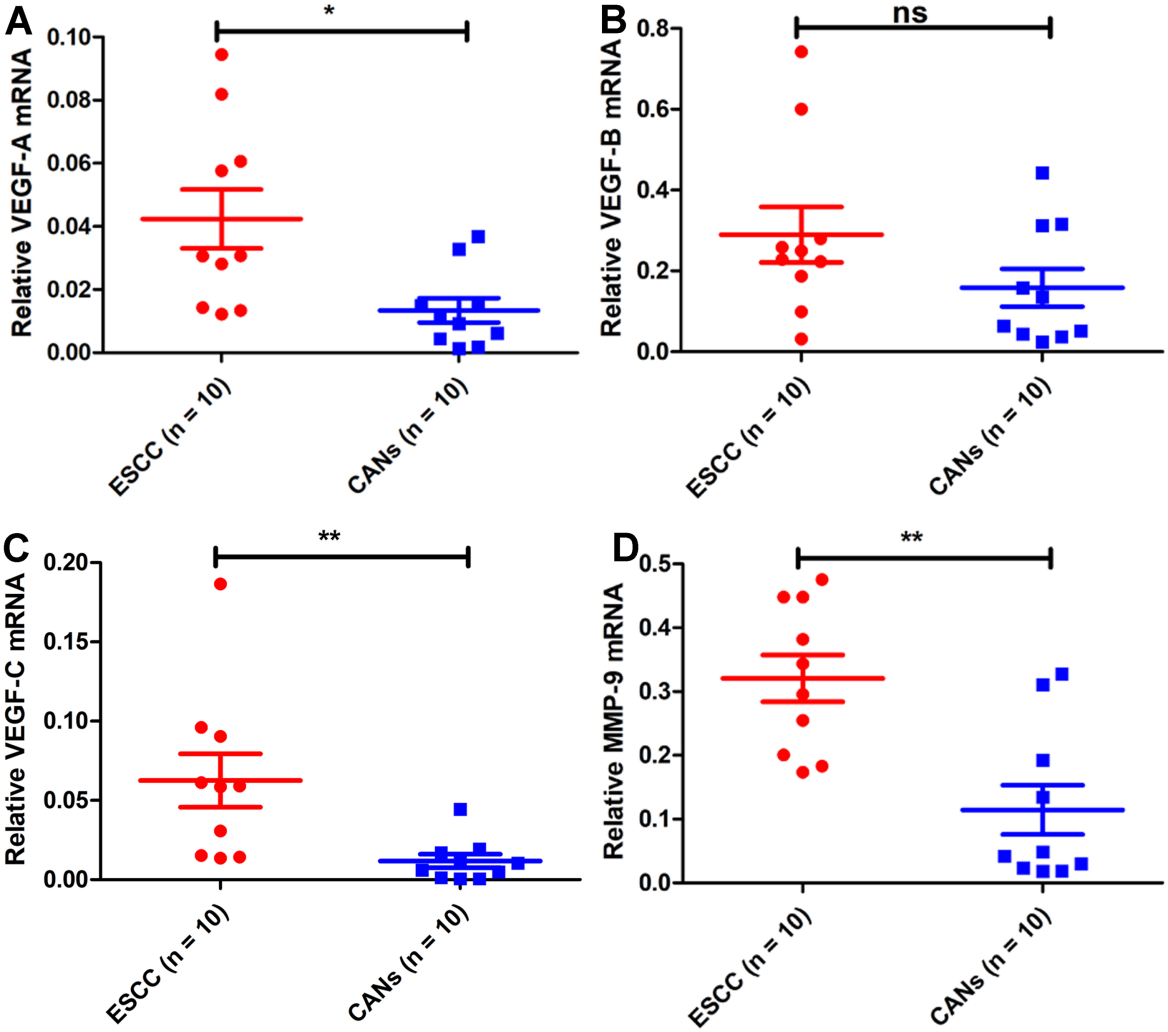
*VEGF* family members and *MMP-9* mRNA expression in samples from Kazakh patients of esophageal squamous cell cancer (ESCC). Compared with that in CANs, *VEGF-A* mRNA was highly expressed (A), *VEGF-B* mRNA expression was not significantly different (B), *VEGF-C* mRNA was highly expressed (C), and *MMP-9* mRNA was highly expressed (D) in samples from Kazakh patients with ESCC. Paired-samples *t* test, *n* = 20, ns *P* > 0.05, *P < 0.05, **P < 0.01, ***P < 0.001.

To further evaluate the findings of the bioinformatics analysis, ten pairs of ESCC tissues and matched CANs from Kazakh patients were selected to find *VEGF* family members and *MMP-9* mRNA expression. *VEGF-A* was highly expressed in Kazakh ESCC tissue samples relative to CANs (Fig. 2A), and there was no obvious difference in *VEGF-B* expression in Kazakh ESCC tissue samples (Fig. 2B). Besides, *VEGF-C* and *MMP-9* were up-regulated in Kazakh ESCC samples than CANs (Figs. 2C–2D). Above all, the Kazakh ESCC tissue samples showed significant overexpression of *VEGF-C* and *MMP-9* mRNA. These results agree with the bioinformatics analysis.

### VEGF-C and MMP-9 protein expression in Kazakh patients with ESCC is associated with progression of ESCC

**Figure 3:**
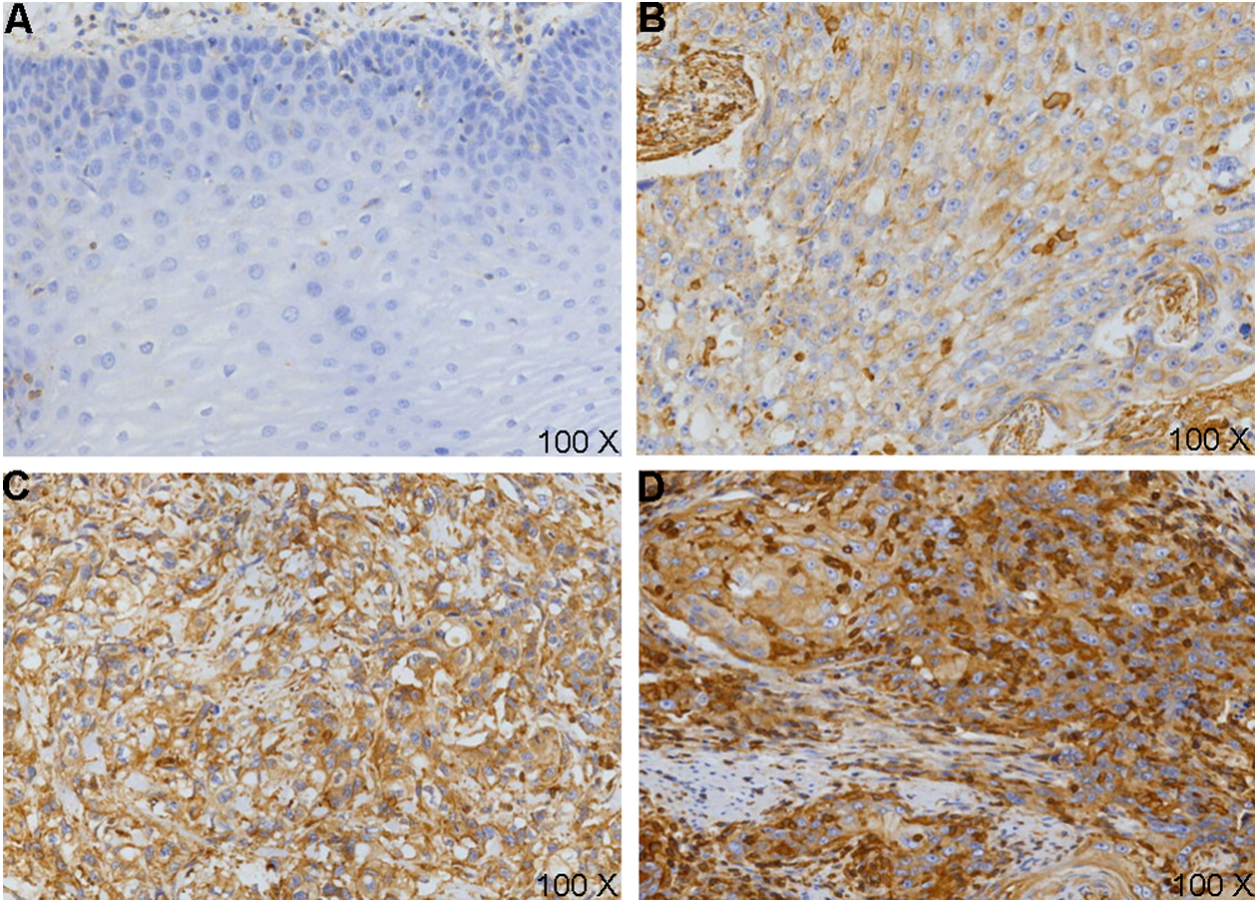
VEGF-C protein expression in esophageal squamous cell carcinoma (ESCC) and cancer adjacent normal (CAN). (A) Loss of expression (0-) of VEGF-C in CANs; VEGF-C positivity expression in cell membranes and cytoplasm, strength as (B) 1 +, (C) 2 +, (D) 3 + in ESCC tissues.

To confirm the above predictive results, IHC was carried out (Fig. 3). We classified the samples depending on VEGF-C protein expression level (0, 1+, 2+, 3+). A signed-rank test demonstrated VEGF-C protein was overexpressed in ESCCs (Table 1).

**Table 1:** The expression of VEGF-C in Kazakh esophageal squamous cell carcinoma (ESCC) and cancer adjacent normal (CAN) tissues.

	**Cases (*N*)**	**0**	**+1**	**+2**	**+3**	**Mean rank**	***Z***	***P*-value**
ESCCs	100	4	24	45	27	123.54	−5.964	<0.001^*^
CANs	100	18	45	35	2	77.47		

**Notes.**

* *P* < 0.05.

For investigate VEGF-C’s effect in Kazakh patients with ESCC, the associated with VEGF-C expression and patients’ clinicopathological factors were studied. We distributed all samples into two subgroups by VEGF-C levels: Negative expression (−0/1+) and positive expression (2+/3+). Compared with males, more female samples’ VEGF-C was expressed (*P* < 0.001). Samples with VEGF-C expression had increased depth of invasion, lymphatic and lymph node metastasis, and worse TNM stage (all *P* < 0.001). No striking relationships were discovered with other features (all *P* > 0.05) (Table 2).

**Table 2:** Correlation between expression of VEGF-C and clinicopathologic parameters in Kazakh esophageal squamous cell carcinoma (ESCC) tissues.

**Variable**	**Cases(*N*)**	**VEGF-C**		***P*-value**
		**Negative (0, 1)**	**Positive (2, 3)**	
Age (*y*)				
≤Median (58 *y*)	52	14	38	0.540
>Median	48	14	34	
Gender				
Male	71	15	56	0.001^*^
Female	29	13	16	
Tumor location				
Upper	1	0	1	0.183
Middle	74	20	54		
Lower	25	8	17	
Histologic grade				
Well	28	4	24	0.183
Moderate	49	16	33		
Poor	23	8	15	
Depth of invasion				
T1–T2	37	16	21	<0.001^*^
T3–T4	63	12	51	
Venous invasion				
Negative	93	27	66	0.399
Positive	7	1	6	
Lymphatic invasion				
Negative	29	16	13	<0.001^*^
Positive	71	12	59	
Nodal status				
pN−	47	22	25	<0.001^*^
pN+	53	6	47	
TNM stage				
I–II	61	26	35	<0.001^*^
III–IV	39	2	37	

**Notes.**

pN− no lymph node metastasis pN + node metastasis

* *P* < 0.05.

Our previous research showed MMP-9 was overexpression in Kazakh ESCCs rather than CANs. We also noted a connection between MMP-9 and lymph node metastasis, depth of invasion, and TNM stage (Hu et al., 2017).

### VEGF-C and MMP-9 are positively and linearly correlated in Kazakh patients with ESCC, and their overexpression predicts increased tumor aggressiveness

**Figure 4:**
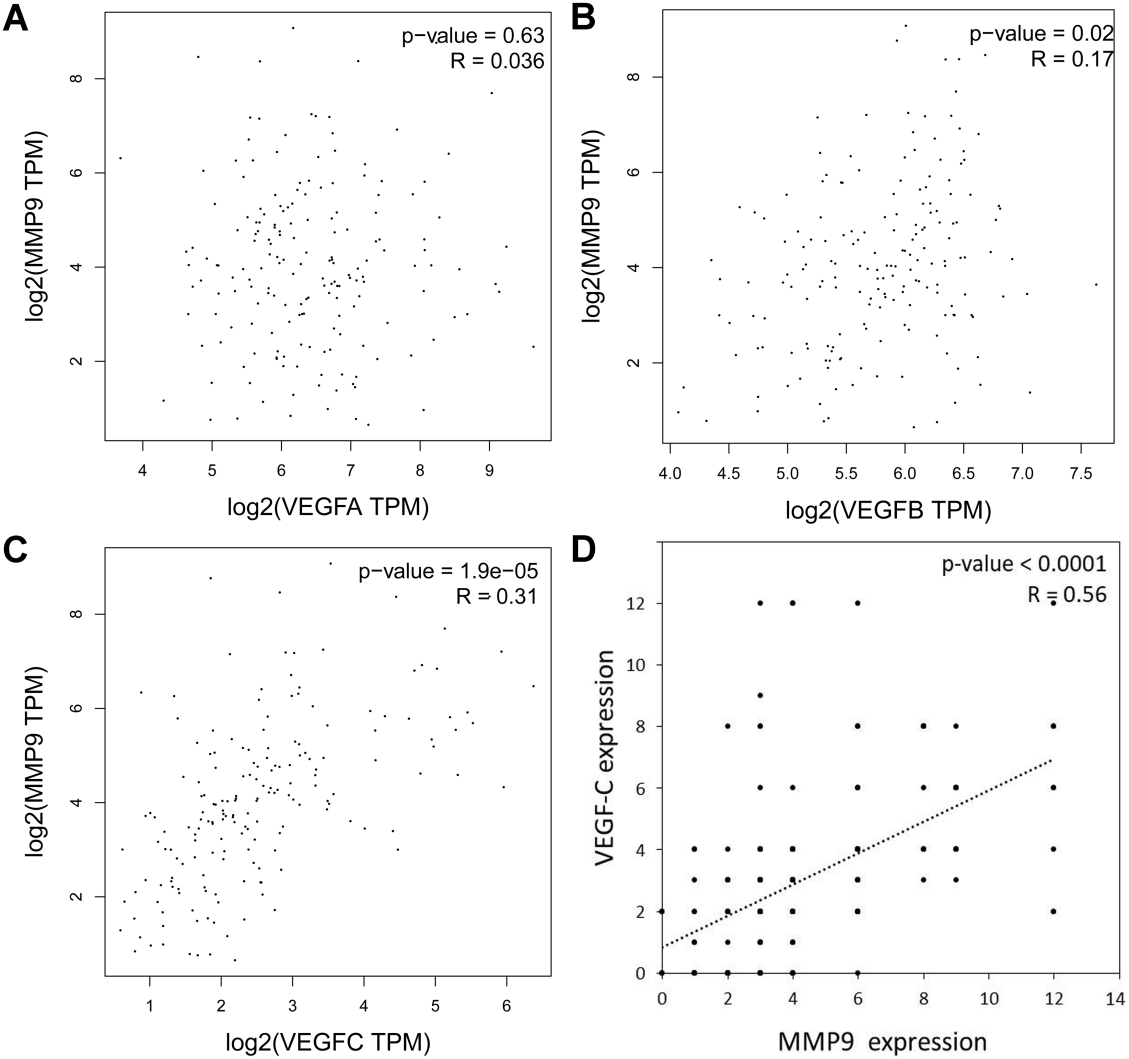
Association with *VEGF* and *MMP-9* in esophageal cancer (EC) and in Kazakh patients with ESCC. (A–C) Correlation analysis of (A) *VEGF-A*, (B) *VEGF-B*, (C) *VEGF-C*, and *MMP-9* mRNA expressions in ECs based on the GEPIA database (including TCGA EC samples *n* = 182), Pearson’s *r* test. (D) Bivariate correlation analyses showing a positive correlation between VEGF-C and MMP-9 expression in ESCC samples from Kazakh patients, *n* = 100, *P* < 0.001, Spearman’s test.

To explore which VEGF family member is most related to MMP-9 expression in EC, we analyzed the correlation between the VEGF family members and MMP-9 according to the TCGA EC samples. In EC, the associated with *VEGF-A* and *MMP-9* mRNA expression was not observed (Fig. 4A), while a weak correlation between *VEGF-B* and it (Fig. 4B), and *VEGF-C* mRNA expression was significantly related to it (Fig. 4C). Consistently, IHC analysis demonstrated a positively and linearly correlated between VEGF-C protein levels and MMP-9 protein levels in samples from Kazakh patients with ESCC (Fig. 4D).

To address whether both VEGF-C/MMP-9 protein expression is involved in the development of ESCC in the Kazakh population, we defined VEGF-C/MMP-9 (0, 1+) as negative expression and VEGF-C/MMP-9 (2+, 3+) as positive expression. We then divided all samples into three subgroups: both expression, single expression, and negative expression of VEGF-C/MMP-9.

Expressed either proteins subgroup included a significantly greater number of cases with a substantial depth of invasion, venous invasion, lymphatic and lymph node metastasis, worse TNM stage and histological grade, relative to the subgroup lacking the expression of both proteins (all *P* < 0.05). No obvious differences in other clinicopathological features (all *P* > 0.05, Table 3). Furthermore, the subgroup that expressed both proteins included a significantly greater number of cases with increased depth of invasion, lymphatic and lymph node metastasis, worse histological grade and TNM stage, relative to the other two subgroups (all *P* < 0.05, Table 4). These data indicated the important role of the overexpression of both proteins in the progression of ESCC in the Kazakh population.

**Table 3:** Correlation between VEGF-C/MMP-9 expression and clinicopathologic parameters in Kazakh esophageal squamous cell carcinoma (ESCC) tissues.

**Variable**	**Cases(*N*)**	**VEGF-C (−)MMP-9 (−)**	**VEGF-C (+)or MMP-9 (+)**	**VEGF-C (+)MMP-9 (+)**	***P*-value**
Age (y)					
≤Median (58 y)	52	13	13	26	0.660
>Median	48	7	17	24	
Gender					
Male	71	13	19	39	0.254
Female	29	7	11	11	
Tumor location					
Upper	1	0	1	0	0.379
Middle	74	14	21	39		
Lower	25	6	8	22	
Histologic grade					
Well	28	4	5	19	0.006^*^
Moderate	49	11	19	19		
Poor	23	5	6	12	
Depth of invasion					
T1–T2	37	12	10	15	0.001^*^
T3–T4	63	8	20	35	
Venous invasion					
Negative	93	19	30	44	0.006^*^
Positive	7	1	0	6	
Lymphatic invasion					
Negative	29	15	7	7	<0.001^*^
Positive	71	5	23	43	
Nodal status					
pN−	47	17	16	14	<0.001^*^
pN+	53	3	14	36	
TNM stage					
I–II	61	19	22	20	<0.001^*^
III–IV	39	1	8	30	

**Notes.**

pN− no lymph node metastasis pN + lymph node metastasis

The Bonferroni method was applied to correct the *P* value.

* *P* < 0.05.

**Table 4:** Stratified analysis of correlation between clinicopathologic features and VEGF-C and MMP-9 expression in Kazakh esophageal squamous cell carcinoma (ESCC) tissues.

**Variable**	***VEGF-C*(−)*MMP-9*(−)**	***VEGF-C*(+)*MMP-9*(+)**	***P*-value**	***VEGF-C*(+)or*MMP-9*(+)**	***VEGF-C*(+)*MMP-9*(+)**	***P*-value**
Histologic grade						
Well	4	19	0.382	5	19	0.001^*^
Moderate	11	19			19		19	
Poor	5	12		6	12	
Depth of invasion						
T1–T2	12	15	<0.001^*^	10	15	0.970
T3–T4	8	35		20	35	
Venous invasion						
Negative	19	44	0.693	30	44	0.005^*^
Positive	1	6		0	6	
Lymphatic invasion						
Negative	15	7	<0.001^*^	7	7	0.067
Positive	5	43		23	43	
Nodal status						
pN^−^	17	14	<0.001^*^	16	14	0.007^*^
pN^+^	3	36		14	36	
TNM stage						
I–II	19	20	<0.001^*^	22	20	<0.001^*^
III–IV	1	30		8	30	

**Notes.**

pN− no lymph node metastasis pN + lymph node metastasis

* *P* < 0.05.

### VEGF-C and MMP-9 protein overexpression predicts poor prognosis in Kazakh ESCCs

**Figure 5:**
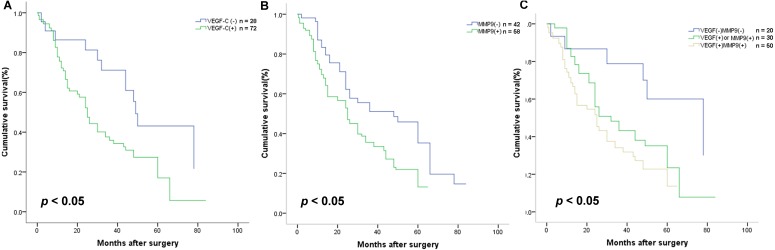
Association with VEGF-C and MMP-9 protein expression and the survival rate of Kazakh patients with esophageal squamous cell cancer (ESCC). Kaplan–Meier survival curves were applied to calculate the relationships. (A) Cases with VEGF-C expression had a shorter overall survival (OS) (*P* = 0.015), *n* = 100. (B) Patients with overexpression of MMP-9 had significantly shorter OS (*P* = 0.005), *n* = 100. (C) The difference in OS between different patterns of VEGF-C and MMP-9 expression (*P* = 0.004), *n* = 100. Compared with patients with both proteins, no expression of protein (*P* = 0.007, *n* = 70) and expressed either protein (*P* = 0.036, *n* = 80) had better OS (log-rank test).

To explore the association of VEGF-C and MMP-9 with ESCC prognosis, Kaplan–Meier survival curves were applied. We found that the OS was significantly shorter in cases with VEGF-C positive expression (*P* < 0.05, Fig. 5A). On the basis of previous studies, it was found MMP-9 level relevance to worse OS in Kazakh patients with ESCC (*P* < 0.05, Fig. 5B).

Different proteins expression types affected the prognosis of Kazakh patients with ESCC (*P* < 0.05, Fig. 5C). Further analysis showed that contrasted with the other subgroups, the cases with expression of both two proteins had worse prognosis (all *P* < 0.05, Fig. 5C).

Univariate analysis, executed according to a Cox proportional hazards model, found that both two proteins expression (Hazard ratio (HR) = 1.760), VEGF-C expression (HR = 2.074), MMP-9 expression (HR = 1.854), depth of invasion (HR = 1.583), lymphatic invasion (HR = 2.569), venous invasion (HR = 2.404), lymph node metastasis (HR = 2.821), and worse TNM stage (HR = 3.449) were factors related to worse outcome of Kazakh ESCCs (all *P* < 0.05). Multivariate analysis found TNM stage is an independent prognostic factor (HR = 2.646, *P* < 0.05) (Table 5).

**Table 5:** Univariate and multivariate analysis of overall survival (OS) of esophageal squamous cell carcinoma (ESCC).

**Variable**	**Cases (*N*)**	**Univariate analysis**			**Multivariate analysis**		
		**HR**	**95% CI**	***P*-value**	**HR**	**95% CI**	***P*-value**
Age (>58 y vs ≤58 y)	100	0.859	0.603–1.225	0.402			
Sex (female vs male)	100	1.029	0.679–1.557	0.894			
Histologic grade (moderate + poor vs well)	100	1.148	0.760–1.734	0.511			
Tumor location (middle vs upper + lower)	100	1.193	0.756–1.885	0.448			
Venous invasion (positive vs negative)	100	3.569	1.624–4.064	<0.001^*^	1.242	0.653–2.363	0.508
Lymphatic invasion (positive vs negative)	100	2.404	1.344–4.297	0.003^*^	1.418	0.768–2.619	0.264
Depth of invasion (T3 + T4 vs T1 + T2)	100	1.583	1.061–2.364	0.025^*^	0.999	0.637–1.567	0.997
Nodal metastasis (+ vs −)	100	2.821	1.897–4.195	<0.001^*^	1.078	0.507–2.292	0.845
TNM stage (III + IV vs I + II)	100	3.449	2.036–5.034	<0.001^*^	2.646	1.379–5.078	0.03^*^
VEGF-C (+ vs −)	100	2.074	1.318–3.700	0.017^*^	1.834	0.718–4.689	0.205
MMP-9 (+ vs −)	100	1.854	1.219–2.820	0.004^*^	2.226	0.670–7.389	0.191
VEGF-C/MMP-9 (both + vs VEGF-C or MMP-9 −)	100	1.760	1.185–2.612	0.005^*^	0.432	0.121–1.548	0.198

**Notes.**

* *P* < 0.05.

## Discussion

The Kazakh minority ethnic population is a special population with the highest incidence of ESCC in worldwide, and its prognosis is worse (Zheng et al., 2010). It is generally considered to be related to the unique genetic background, environmental factors, and eating habits of the Kazakh minority ethnic population (Wang et al., 2010). Early invasion and metastasis are one of major reasons for poor prognosis in patients with ESCC (Cools-Lartigue, Spicer & Ferri, 2015). Tumor vascular formation plays critical role in tumor infiltration (Skobe et al., 2001; Weidner et al., 1991). Thus, promote angiogenesis and lymphangiogenesis factors in the tumor microenvironment have received increased research attention. Inhibiting tumor angiogenesis and lymphangiogenesis, and disrupting the microenvironment required for tumor growth, which are important directions for anti-tumor therapy (Li et al., 2018; Shojaei, 2012).

The VEGF family contains many members (Clauss, 2000). Among them, VEGF-A and VEGF-C are abnormally up-regulation in EC, in which they are related to worse prognosis (Juchniewicz et al., 2015; Kimura et al., 2008; Kozłowski et al., 2013). *VEGF-A* and *VEGF-C* mRNA were up-regulated in EC, whereas overexpression of VEGF-C in ESCC was more significant in this research. It was confirmed in Kazakh patients with ESCC. There are obvious differences in the expression of *VEGF* family members in ESCC and EAC, because they arise from different cell populations (Chang & Katzka, 2004). Therefore, the molecular genetic background of ESCC and EAC are different (Agrawal et al., 2012). VEGF-C is related to lymphatic metastasis rather than vascular metastasis in Kazakh ESCCs. Lymph node invasion is the major way of infiltration of EC and a critical cause of poor prognosis (Argyres, 2004; Schiefer, Schoppmann & Birner, 2016). Consistent with our analysis, VEGF-C could play a crucial role in lymphangiogenesis in ESCC, but not in EAC (Möbius et al., 2007). VEGF-C promotes esophageal lymphangiogenesis, which may be a crucial factor in the early invasion and metastasis in Kazakh patients with ESCC.

MMP-9 is generally regarded as a prognostic indicator for EC (El-Kenawy et al., 2005). In the study of ESCC, MMP-9 is overexpression in more malignant tumors with lymph node invasion (Ohashi et al., 2000; Samantaray et al., 2004). We found MMP-9 is overexpression and related to increased lymph node metastasis in Kazakh ESCCs in our research at a previous time (Hu et al., 2017).

VEGF-C/MMP-9 was substantially involved in the early metastasis of ESCC. However, there have been no reports on the simultaneous investigation of them. It is necessary to study them simultaneously. In this work, we analyzed the associated with VEGF and MMP-9 in EC. We noted a positive linear relationship between *VEGF-B*, *VEGF-C*, and *MMP-9* in EC by TCGA database. Compared to *VEGF-B*, *VEGF-C* is more related to *MMP-9*; therefore, we chose VEGF-C for further study. Our analysis showed the same result in Kazakh ESCC samples, as noted in EC samples. MMP-9 and VEGF-C play a coordinating role in neck squamous cell cancer progression, in which VEGF-C overexpression stimulates excessive secretion of MMP-9 (Deryugina, Liliana & Strongin, 2002). VEGF-C and MMP-9 have potential synergistic effects in Kazakh patients with ESCC, promoting early tumor invasion and metastasis. There has been no research on the synergistic effect of VEGF-B and MMP-9 in ESCC, which will form the basis for our future investigations.

Our results showed that cases with overexpression of both VEGF-C/MMP-9 had a significantly greater depth of invasion, lymphatic and lymph node metastasis, worse histological grade and TNM stage. Interestingly, patients overexpressing both two proteins not only had more lymph node and lymphatic metastasis, but also had higher levels of vascular metastasis. The combination of two proteins could predict tumor vascular metastasis. Tumor angiogenesis and lymphangiogenesis are dynamic processes that require the activation of MMPs mediated by VEGF (especially MMP-9) to induce the release of soluble KIT ligands (sKitL) (Rafii et al., 2002). SKitL promotes the proliferation and movement of EPCs and hematopoietic cells in the bone marrow microenvironment, laying the foundation for their mobilization to the peripheral circulation (Rafii et al., 2002).

Regarding the VEGF-C-MMP-9 signaling pathway, VEGF-C is expressed in cancer cells, lymphatic vessels, and vascular endothelial cells, as well as in macrophages (Takizawa et al., 2006). VEGF-C mainly in association with VEGF receptor-2 (VEGFR-2) and VEGFR-3, to promote lymphangiogenesis (Joukov et al., 1996). MMP9 in the tumor microenvironment provides the conditions for tumor metastasis in a manner dependent on VEGFR-1 (Kempen & Coussens, 2002). Notch signaling activates downstream of VEGF, promotes the secretion of MMP-9, and then induces blood vessel endothelial cell formation (Funahashi et al., 2011). Importantly, the induction of VEGF and MMP9 expression by macrophages from the tumor microenvironment is also one of the important sources (Li et al., 2012). Tumor angiogenesis and lymphangiogenesis are complex processes with multiple links. In addition to the involvement of the VEGF family and MMP9, a variety of factors are involved in these processes, such as fibroblast growth factor (FGF), angiopoietin (ANG), and hypoxia-inducible factor-1 (HIF-1) (Koblizek et al., 1998; Maxwell et al., 1997; Presta et al., 2005). The association with these factors and the progress of Kazakh ESCCs will be the direction of our subsequent research.

Compared with single protein expression, the prognosis of Kazakh patients with ESCC with both two proteins expression was worse. Thus, the combined application of two proteins has prognostic value.

## Conclusions

Bioinformatic analyses showed that *VEGF-C* and *MMP-9* are associated with ESCC. Both of them promote tumor pathological vascular and lymphatic vessel formation, providing preconditions for infiltration and metastasis of ESCC in the Kazakh population. The up-regulated of both two proteins was correlated with shorter OS of Kazakh ESCCs. The synergistic effect of the two proteins could be used to diagnose ESCC in the Kazakh population and has a better suggestive effect. Targeting both VEGF-C and MMP-9 could be an important direction for the treatment of ESCC in the Kazakh population.

##  Supplemental Information

10.7717/peerj.8182/supp-1 Table S1 The characteristic of 100 cases of Kazakh patients with esophageal squamous cell carcinoma (ESCC)

10.7717/peerj.8182/supp-2 Table S2 Primer sequences of target genes

10.7717/peerj.8182/supp-3 Figure S1 The difference expression of VEGF family and MMP-9 in esophageal adenocarcinoma (EAC) according to bioinformatics analysis Comparison of (A) VEGF-A, (B)VEGF-B, (C)VEGF-C and (D)MMP-9 expression in EAC tissues and normal tissues in according to the Oncomine database. *n* = 16, Student’s *t*-test.

10.7717/peerj.8182/supp-4 Supplemental Information 1 qRT PCR raw data

10.7717/peerj.8182/supp-5 Supplemental Information 2 IHC raw data

## Additional Information and Declarations

### Data Availability

The following information was supplied regarding data availability: The raw measurements are available in the Supplemental Files.
