# Supplementary material for: Overexpression of VEGF-C and MMP-9 predicts poor prognosis in Kazakh patients with esophageal squamous cell carcinoma
Source: PeerJ. 2019 Dec 3;7:e8182. doi: 10.7717/peerj.8182 (PMC6896941; doi:10.7717/peerj.8182)
Supplement: Table S1 [file peerj-07-8182-s001.docx]

**Supplementary Table 1** The characteristic of 100 cases of Kazakh patients with esophageal squamous cell carcinoma (ESCC).

|  | **Variable** | **Cases (N)** |
| --- | --- | --- |
| Age (y) | ≤ Median (58y) | 52 |
|  | > Median | 48 |
| Gender | Male | 71 |
|  | Female | 29 |
| Tumor location | Upper | 1 |
|  | Middle | 74 |
|  | Lower | 25 |
| Histologic grade | Well | 28 |
|  | Moderate | 49 |
|  | Poor | 23 |
| Depth of invasion | T1 | 4 |
|  | T2 | 33 |
|  | T3 | 43 |
|  | T4 | 20 |
| Venous invasion | Negative | 93 |
|  | Positive | 7 |
| Lymphatic invasion | Negative | 29 |
|  | Positive | 71 |
| [Nodal](http://dict.cnki.net/hot_eng/dict_source.aspx?searchword=nodal) status | No lymph node metastasis | 47 |
|  | Lymph node metastasis | 53 |
| TNM stage | I-II | 61 |
|  | III-IV | 39 |

**Supplementary Table 2** Primer sequences of target genes.

| **Target** | **Sense Primer** | **Antisense Primer** |
| --- | --- | --- |
| VEGF-A | 5′-ACGAACGTACTTGCAGATGTG-3′ | 5′-TTCTGTCGATGGTGATGGTGT-3′ |
| VEGF-B | 5′- GAAAGTGGTGTCATGGATAG3-3′ | 5′- ATGAGCTCCACAGTCAAG3-3′ |
| VEGF-C | 5′-CAGCAAGACGTTGTTTGAAATTACA-3′ | 5′-GTGATTGGCAAAACTGATTGTGA-3′ |
| MMP-2 | 5′-CCACTGCCTTCGATACAC-3′ | 5′-GAGCCACTCTCTGGAATCTTAAA-3′ |
| MMP-9 | 5′- GTTCCCGGAGTGAGTTGA -3′ | 5′- TTTACATGGCACTGCCAAAGC -3′ |
