# Supplementary material for: Overexpression of VEGF-C and MMP-9 predicts poor prognosis in Kazakh patients with esophageal squamous cell carcinoma
Source: PeerJ. 2019 Dec 3;7:e8182. doi: 10.7717/peerj.8182 (PMC6896941; doi:10.7717/peerj.8182)
Supplement: Table S2 [file peerj-07-8182-s002.docx]

**Supplementary Table 2** Primer sequences of target genes.

| **Target** | **Sense Primer** | **Antisense Primer** |
| --- | --- | --- |
| VEGF-A | 5′-ACGAACGTACTTGCAGATGTG-3′ | 5′-TTCTGTCGATGGTGATGGTGT-3′ |
| VEGF-B | 5′- GAAAGTGGTGTCATGGATAG3-3′ | 5′- ATGAGCTCCACAGTCAAG3-3′ |
| VEGF-C | 5′-CAGCAAGACGTTGTTTGAAATTACA-3′ | 5′-GTGATTGGCAAAACTGATTGTGA-3′ |
| MMP-2 | 5′-CCACTGCCTTCGATACAC-3′ | 5′-GAGCCACTCTCTGGAATCTTAAA-3′ |
| MMP-9 | 5′- GTTCCCGGAGTGAGTTGA -3′ | 5′- TTTACATGGCACTGCCAAAGC -3′ |
