# Supplementary figures and images for: Overexpression of VEGF-C and MMP-9 predicts poor prognosis in Kazakh patients with esophageal squamous cell carcinoma
Source: PeerJ. 2019 Dec 3;7:e8182. doi: 10.7717/peerj.8182 (PMC6896941; doi:10.7717/peerj.8182)

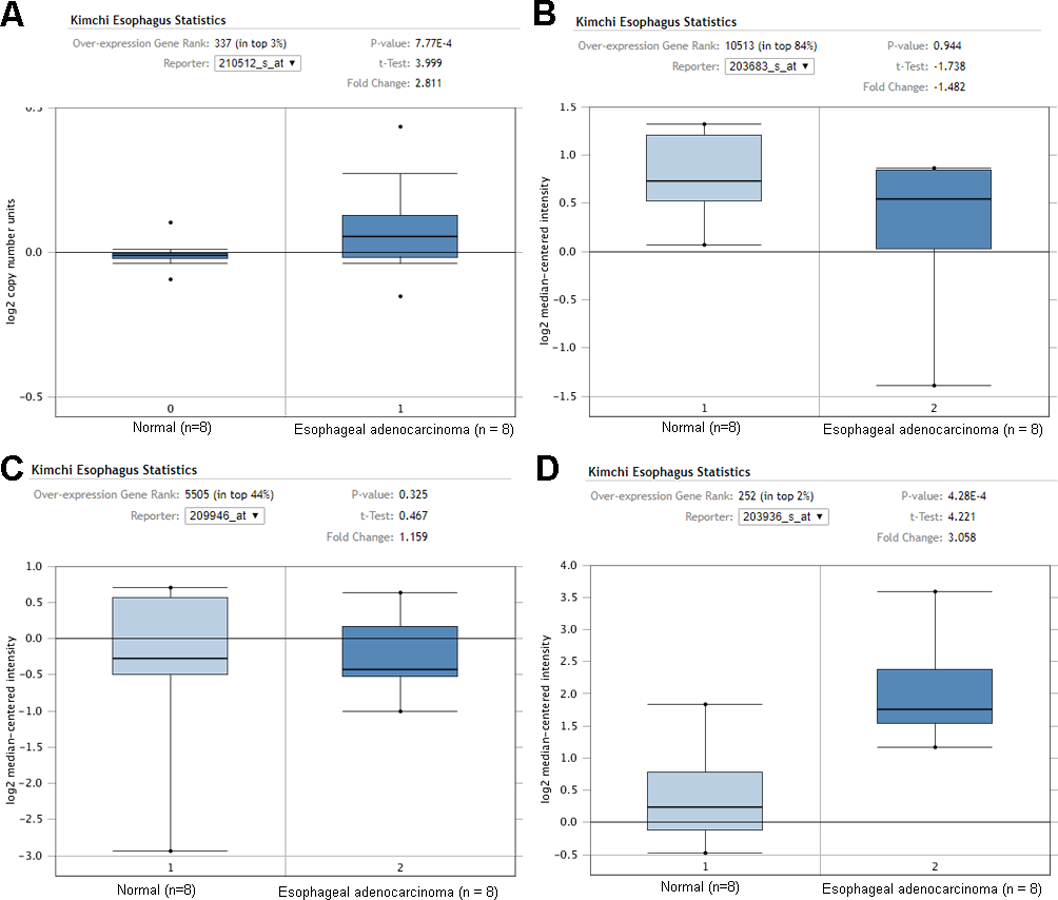

Supplement: Figure S1 — Comparison of (A) VEGF-A, (B)VEGF-B, (C)VEGF-C and (D)MMP-9 expression in EAC tissues and normal tissues in according to the Oncomine database. n = 16, Student’s t-test. [file peerj-07-8182-s003.png]
